# Error Modeling and Experimental Study of a Flexible Joint 6-UPUR Parallel Six-Axis Force Sensor

**DOI:** 10.3390/s17102238

**Published:** 2017-09-29

**Authors:** Yanzhi Zhao, Yachao Cao, Caifeng Zhang, Dan Zhang, Jie Zhang

**Affiliations:** 1 Key Laboratory of Parallel Robot and Mechatronic System of Hebei Province, Yanshan University, Qinhuangdao 066004, China; yccaoryan@stumail.ysu.edu.cn (Y.C.); cfzhang@stumail.ysu.edu.cn (C.Z.); 2 Key Laboratory of Advanced Forging & Stamping Technology and Science of Ministry of Education of China, Yanshan University, Qinhuangdao 066004, China; 3 Department of Mechanical Engineering, Lassonde School of Engineering, York University, 4700 Keele Street, Toronto, ON M3J1P3, Canada; dan.zhang@lassonde.yorku.ca; 4 Department of Basic Teaching, LiRen College of Yanshan University, Qinhuangdao 066004, Hebei, China; jiezhang@ysu.edu.cn

**Keywords:** parallel six-axis force sensor, flexible joints, error modeling, Monte Carlo method, calibration experiment

## Abstract

By combining a parallel mechanism with integrated flexible joints, a large measurement range and high accuracy sensor is realized. However, the main errors of the sensor involve not only assembly errors, but also deformation errors of its flexible leg. Based on a flexible joint 6-UPUR (a kind of mechanism configuration where U-universal joint, P-prismatic joint, R-revolute joint) parallel six-axis force sensor developed during the prephase, assembly and deformation error modeling and analysis of the resulting sensors with a large measurement range and high accuracy are made in this paper. First, an assembly error model is established based on the imaginary kinematic joint method and the Denavit-Hartenberg (D-H) method. Next, a stiffness model is built to solve the stiffness matrix. The deformation error model of the sensor is obtained. Then, the first order kinematic influence coefficient matrix when the synthetic error is taken into account is solved. Finally, measurement and calibration experiments of the sensor composed of the hardware and software system are performed. Forced deformation of the force-measuring platform is detected by using laser interferometry and analyzed to verify the correctness of the synthetic error model. In addition, the first order kinematic influence coefficient matrix in actual circumstances is calculated. By comparing the condition numbers and square norms of the coefficient matrices, the conclusion is drawn theoretically that it is very important to take into account the synthetic error for design stage of the sensor and helpful to improve performance of the sensor in order to meet needs of actual working environments.

## 1. Introduction

Compared with the traditional multi-axis force sensor, the sensor with flexible joints has advantages of fast response, small accumulated error, no mechanical friction and high measurement accuracy, so it has broad application prospects [1,2,3,4,5]. At present, the design of sensors with flexible joints can be divided into two categories: the majority of sensors are designed and processed based on the integral structure. The other is using the assembled structure. For the former, there have been numerous research achievements. Kerr [6] proposed that the Stewart platform with instrumented elastic legs can be used as a six-axis force sensor. Gao et al. [7] developed a six-axis controller based on the Stewart platform-based force sensor, and introduced the use of elastic joints to replace the real spherical joints which made miniaturization possible. Liang et al. [8] designed and developed a new six-axis sensor system with a compact monolithic elastic element, which detected the tangential cutting forces along the *x*-, *y*-, and *z*-axes as well as the cutting torques about the *x*-, *y*-, and *z*-axes simultaneously. Unfortunately, restricted by their integrated structure, most of the sensors mentioned above are used in a small range of applications. In addition, the main error source of these sensors is deformation error. As for the latter assembled by flexible kinematic joints, Yang [9] developed a planar three-axis force sensor with flexible joints to diagnose and monitor bearing faults online in real time. Zhang [10] studied the model reconstruction theory of flexible assembly six-axis force sensors based on a hybrid leg spoke layout. Li [11] established an integral stiffness model of a flexible assembly six-axis force sensor based on the Stewart mechanism. These sensors are assembled traditionally. Consequently, the errors are mainly caused by the assembly process, which leads to large errors and low accuracy, so how to achieve high accuracy while taking into account a large measurement range is still a challenging problem. At present, there is limited literature available on this issue. Zhao et al. [12] proposed a large measurement range flexible joints six-axis sensor. Its mathematical modeling and calibration experiments were performed.

Inevitably, the main errors of flexible assembly force sensors involve not only deformation errors, but also assembly errors. Many excellent studies [13,14,15,16] on error modeling and analysis of the parallel mechanism have been conducted so far. Arai and Ropponen [17] modeled and analyzed the error of the Stewart mechanism based on the vector algebra loop increment method. In addition, through the singular value decomposition of the force Jacobian, analytical expressions of the structural parameters of the Stewart platform, actuated error and end error were obtained. Wang and Massory [18,19] introduced the joint point error and actuated joint error, and end error of the mechanism was solved by a D-H numerical method. Wang and Ehmann [20] used a coordinate transformation method to establish input-output equations including the joint manufacturing error and positioning error, and then directly differentiated it, establishing the error model. Aimed at manufacturing error, installation error and actuator motion error of the parallel mechanism, Patel and Ehmann [21] performed an error modeling and analysis of a parallel machine in terms of route planning by means of a mechanism motion differential method and further considered the effect of joint manufacturing errors on end pose. Zou et al. [22] quantitatively analyzed the influence of characteristic parameter errors on the end pose error of the mechanism by using the error transfer matrix of the parallel mechanism. Huang [23] applied screw theory to model and analyze known size errors, control errors and kinematic joint gap errors. Ma et al. [24] established a space vector chain model and deduced the analytic mapping relationship between manufacturing errors of a parallel machine and the pose error of a moving platform. Lv et al. [25] proposed an error modeling method based on the forward kinematics problem. Unfortunately, there are few related literatures that comprehensively consider modeling the two main types of error (assembly error and deformation error), which results in some limitations to improve accuracy of large measurement range sensors.

Based on the flexible joints 6-UPUR six-axis force sensor developed in the prephase, this paper focuses on establishment of the error modeling, namely, assembly error modeling and deformation error modeling. The synthetic error of the force-measuring platform is superposed by the two kinds of errors, resulting in a total pose error. Then, the corresponding first order influence coefficient matrix $G^{\prime }$ is calculated. Meanwhile, deformation of the force-measuring platform are detected by using laser interferometry and analyzed to verify the correctness of the sensor error model, and calibration experiments are completed to obtain the first order kinematic influence coefficient matrix $G_{B}^{\prime }$ in actual circumstances.

The structure of this paper is as follows: after this Introduction, Section 2 introduces the structure of the prototype sensor and solves the theoretical first order kinematic influence coefficient. Section 3 and Section 4 present the error modeling and analysis of the sensor in terms of assembly error and deformation error, respectively. Section 5 comprehensively considers the two main errors, and the first order kinematic influence coefficient when the synthetic error is taken into account is obtained. Section 6 introduces the experimental research on measurement and calibration of the sensor prototype and analyzes the results of the experiment. The paper is concluded in Section 7, summarizing the work that has been done.

## 2. Prototype of the Flexible Joints 6-UPUR Six-Axis Force Sensor

**Figure 1:**
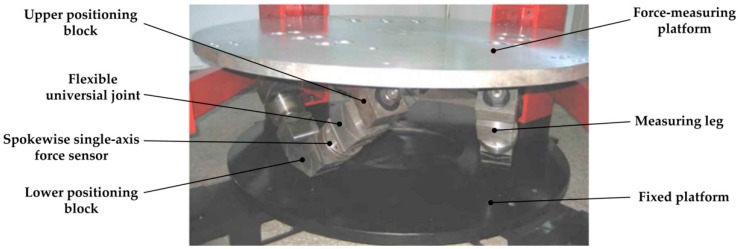
Physical prototype of the 6-UPUR six-axis force sensor with flexible joints.

A physical prototype of the large measurement range 6-UPUR six-axis force sensor with flexible joints was manufactured, as shown in Figure 1. Considering the manufacturing process and economic cost, the material properties of the sensor are listed in Table 1. The main parameters of the sensor are as follows: radius of the force-measuring platform is 550 mm; radius of the fixed platform is 550 mm; the vertical distance between the two platforms is 300 mm; measuring range are: F_x_: ±10,000 N, F_y_: ±10,000 N, F_z_: ±10,000 N, M_x_: ±5,000 N m, M_y_: ±5,000 N m, M_z_: ±5,000 N m and overload capacity is 120%.

**Figure 2:**
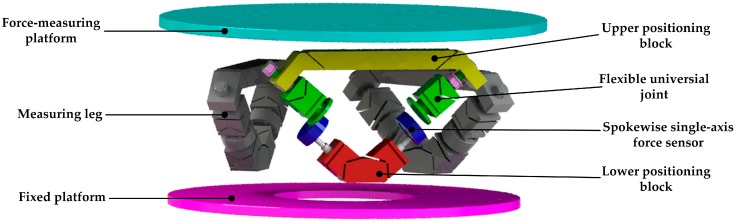
3D Model of the 6-UPUR six-axis force sensor with flexible joints.

A 3D model of the six-axis force sensor with flexible joints is shown in Figure 2. The structure where all joints are flexible joints with a single degree of freedom is adopted. Each leg is a split structure. The upper positioning block is composed of two flexible rotation joints, and one of the joints forms a flexible spherical joint with the flexible universal joint by an assembling relationship. The middle part of the leg is mounted by a single-axis force sensor. The lower part is composed of a flexible universal joint with an integral structure and a lower positioning block. Each elastic leg is connected to the measuring-force and fixed platforms through the upper and lower positioning blocks by bolts, respectively. Thus, decomposition of the six-axis external force to the six legs is realized.

**Figure 3:**
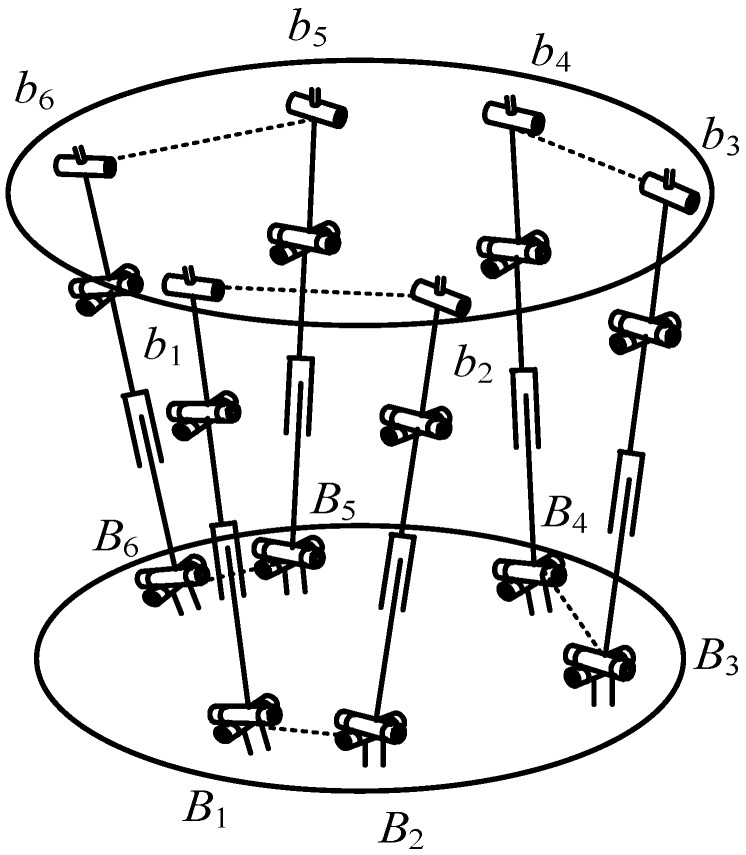
The force sensor structure based on 6-UPUR parallel mechanism.

Figure 3 illustrates the sensor structure based on 6-UPUR parallel mechanism. $B_{i}\left(i=1,2,\cdots ,6\right)$ stands for center point of the first revolute joint axis on the lower positioning block, which is adjacent to the fixed platform. $b_{i}\left(i=1,2,\cdots ,6\right)$ denotes center point of the revolute joint axis on the upper positioning block. Their coordinate matrices are expressed as $r_{B}$ and $r_{b}$, respectively. According to space static equilibrium conditions, the following equation can be obtained by screw theory [26]:

(1) $$F_{w}=\sum_{i=1}^{6}f_{a}^{i}{\$}_{i}$$

where $f_{a}^{i}$ represents magnitude of axial tension/compression force on the *i*-th measuring leg; ${\$}_{i}$ represents the unit line vector along the *i*-th measuring leg, expressed as ${\$}_{i}={\left(\begin{array}{cc} S_{i} & S_{0i} \end{array}\right)}^{T}$; $F_{w}$ is referred to generalized external force vector on center of the measuring platform, expressed as $F_{w}={\left(\begin{array}{cc} f_{w} & m_{w} \end{array}\right)}^{T}$, then, it can be obtained as:

(2) $$\{\begin{array}{l} f_{w}=\sum_{i=1}^{6}f_{a}^{i}S_{i}\\ m_{w}=\sum_{i=1}^{6}f_{a}^{i}S_{0i} \end{array}$$

where $S_{i}=\left[r_{b}\left(:i\right)-r_{B}\left(:i\right)\right]/\left|r_{b}\left(:i\right)-r_{B}\left(:i\right)\right|$; $S_{0i}=\left[r_{b}\left(:i\right)\times r_{B}\left(:i\right)\right]/\left|r_{b}\left(:i\right)-r_{B}\left(:i\right)\right|$.

Then, Equation (1) can be rewritten in form of matrix expression as:

(3) $$F_{w}=GF_{a}$$

where $F_{a}$ represents axial tension/compression force of all legs, expressed as $F_{a}={\left(\begin{array}{llllll} f_{a}^{1} & f_{a}^{2} & f_{a}^{3} & f_{a}^{4} & f_{a}^{5} & f_{a}^{6} \end{array}\right)}^{T}$; G denotes the first order kinematic influence coefficient matrix which is also called Jacobian matrix:

(4) $$G=\left[\begin{array}{llll} S_{1} & S_{2} & \cdots & S_{6}\\ S_{01} & S_{02} & \cdots & S_{06} \end{array}\right]$$

The Jacobian matrix directly determines many characteristics of the sensor, such as tis isotropy, stiffness, sensitivity, etc. It is the foundation to study the performance and structure design of the sensor.

## 3. Assembly Error Modeling of the 6-UPUR Force Sensor Based on Imaginary Kinematic Joint Method

In the last section, the Jacobian matrix G between the six-axis external force exerted on the sensor and axial tension/compression force on the measuring legs is a definite value. But in practice due to the deformation caused by manufacturing, assembly and calibration, the mechanical part will suffer a certain deviation. Thus, the transformation relation in different coordinate frames of the sensor is changed, which leads to a change of the originally set sensor working position and forms a measurement error. Consequently, in this section the assembly error of the 6-UPUR parallel six-axis force sensor is modeled. This part mainly aims at radius errors of the force-measuring platform and fixed platform, errors of two axial clearances for the lower positioning block and the middle universal joint and installation error of single-axis force sensor. The deformation error model of the sensor is established in the next section.

**Figure 4:**
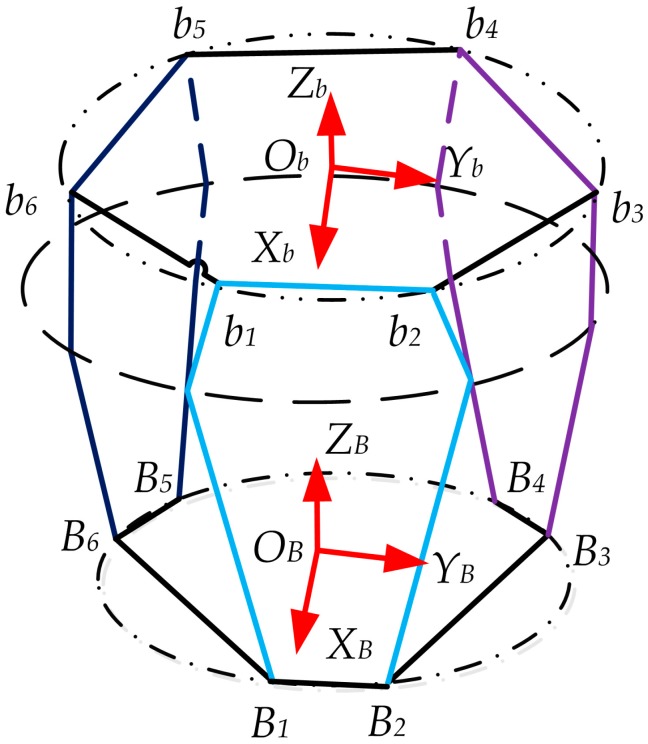
Diagram of fixed coordinate frame and moving coordinate frame of the sensor structure.

The working position error of the force-measuring platform is accumulated by the five errors of one corresponding leg. To establish the sensor error model easily, the fixed coordinate frame and moving coordinate frame are defined as shown in Figure 4.

$B_{i}\left(i=1,2,\cdots ,6\right)$ stands for center point of the first revolute joint axis on the lower positioning block, which is adjacent to the fixed platform. These six points can theoretically compose a planar hexagon. A fixed coordinate frame named $O_{B}-X_{B}Y_{B}Z_{B}$ is attached to the geometric center point $O_{B}$ of the hexagon. The $Z_{B}$-axis is arranged on the normal direction of the fixed base plane; the $X_{B}$-axis is perpendicular to connection between two points $B_{1}$ and $B_{2}$; the $Y_{B}$-axis is determined by the right-hand rule. Similarly, $b_{i}\left(i=1,2,\cdots ,6\right)$ stands for center point of the revolute joint axis on the upper positioning block, and a moving coordinate frame $O_{b}-X_{b}Y_{b}Z_{b}$ is established.

**Figure 5:**
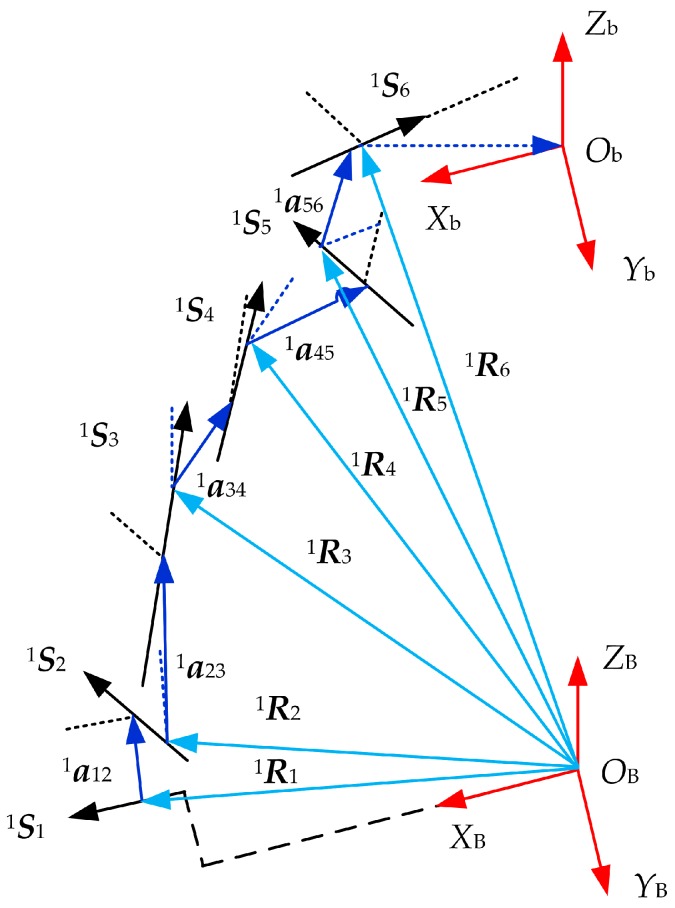
D-H coordinate frame of the *i*-th leg.

Applying the D-H method [27], we establish a local coordinate frame on the *i*-th measuring leg as shown in Figure 5. Sji, aj(j+1)i respectively refer to the axial vector of the *j*-th link on the *i*-th leg and common normal line vector between two adjacent axes, which can be expressed as:

(5) Sji=Tj−1[0−sinα(j−1)jicosα(j−1)ji]

(6) aj(j+1)i=Tj−1[cosθjicosα(j−1)jisinθjisinα(j−1)jisinθji]

where Tji denotes rotation transform matrix of a local coordinate frame of the *j*-th link on the *i*-th leg relative to the fixed coordinate frame $O_{B}-X_{B}Y_{B}Z_{B}$, which can be obtained as:

(7) Tji=[aj(j+1)Sj×aj(j+1)Sj]

The setover along Sji of two adjacent common normal line a(j−1)ji and aj(j+1)i is denoted by Sji. The length of the common normal line and rotation angle are denoted by aj(j+1)i and θji, respectively.

**Figure 6:**
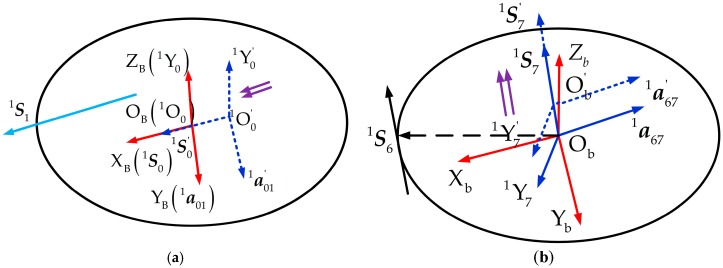
Diagram of imaginary prismatic joints. (**a**) Coordinate frame establishment of the fixed platform radius error; (**b**) Coordinate frame establishment of the measuring platform radius error.

As is well known, S1i represents the axis of the revolute joint. If there exists rotation around the $X_{B}$-axis, it can directly map to S1i. However, if there exists translation along the $X_{B}$-axis, that is to say, the radius error of the fixed platform is taken into account, it will lack certain definition. For this purpose, a new error modeling mechanism method is proposed. That is, the radius error of a fixed platform is represented by an imaginary prismatic joint which is mounted on the connection between the leg and the fixed platform. We define its motion along positive half of the $X_{B}$-axis as the positive direction, namely, there exists a positive radius error, and the corresponding coordinate frame OB−a01iY0iS0i is established. By the same reason, the radius error of the force-measuring platform is also represented by an imaginary prismatic joint and the corresponding coordinate frame Ob−a67iY7iS7i is established. These imaginary prismatic joints and coordinate frames are illustrated in Figure 6.

Rji denotes the position vector of the origin Oji of the *j*-th link on the *i*-th leg expressed in the fixed coordinate frame. It can be calculated by the following equations,

(8) Rji=S1iS1i+a12ia12i+S2iS2i+a23ia23i+⋯+SjiSji

Pi denotes position vector of the origin $O_{b}$ of the force-measuring platform expressed in the fixed coordinate frame. It can be obtained using the following equation:

(9) Pi=S1iS1i+a12ia12i+⋯+S5iS5i+a56ia56i+S6iS6i+a67ia67i+S7iS7i

According to Equations (5)–(9) and combining the kinematic influence coefficient theory, the rotation influence coefficient sub-matrix G3×7Ri and translation influence coefficient sub-matrix G3×7Pi of each legs can be solved. For general parallel mechanisms, the following relationship exists between the matrices G3×7Ri, G3×7Pi and parameters ai, Si, θi, αi [28]:

(10) {∂GPi∂aji=Ga jPi∂GPi∂Sji=Gs jPi∂GPi∂θji=Gθ jPi,∂GRi∂θji=Gθ jRi∂GPi∂αji=Gα jPi,∂GRi∂αji=Gα jRi

Then, all the corresponding influence coefficient matrices GaPi, GSPi, GθRi, GθPi, GαRi and GαPi of each error source can be solved by Equation (10).

**Figure 7:**
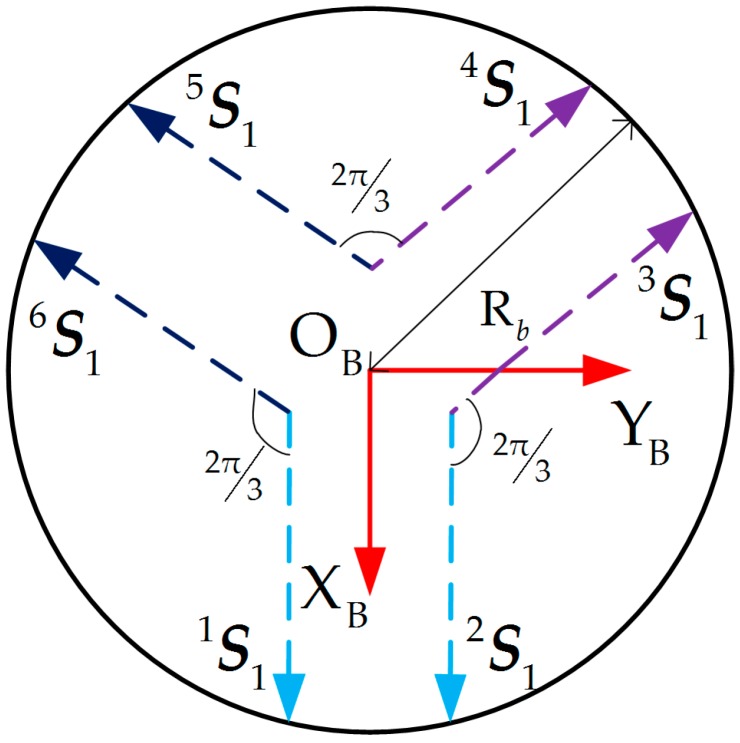
Diagram of equivalent angles.

Due to existence of the actual assembly errors, vectors S1i, S2i, S3i, S4i, S5i, and S6i are not coplanar. By the space geometry and sensor accuracy requirements, S1i(i=1,2,⋯,6) can be assumed in the plane $X_{B}Y_{B}$, as shown Figure 7, as is the axial vector S6i(i=1,2,⋯,6) of the revolute joint on the upper positioning block.

Taking S61 for example, according to the design and processing requirements of the sensors, the directions of S61 and S31 are identical. Meanwhile, S31 is taken as the direction that joint $b_{1}^{\prime }$ points at joint $B_{1}$, i.e.:

(11) S31=(b1′−B1)|b1′−B1|

S11 and S21 represent both axes of the universal joint on the lower positioning block, so they meet the relationship:S21=S11×S31. From the structure of the sensor, it can be seen that S4i, S1i and S2i, S5i are respectively in same direction due to identical direction of the two universal joints. So far, all the axis vectors on the first measuring leg have been found out, and the other vectors can be obtained by the same way.

Then, twist angles of all axes can be obtained as: α01i=0∘, α12i=3π2, α23i=π2, α34i=π2, α45i=π2, α56i=3π2, α67i=0∘. Meanwhile, other D-H parameters are further obtained by the following equation:

(12) {aj(j+1)i=Sji×S(j+1)icosθji=(aj(j+1)i×a(j−1)ji)|aj(j+1)i×a(j−1)ji|

Consequently, the error influence coefficients of each leg, including rotation influence coefficient G3×7Ri and translation influence coefficient G3×7Pi will be calculated according to kinematic influence coefficient theory [26] after the D-H coordinate frame of the *i*-th leg is established.

Error integrations of each leg can be expressed as in vector form: Δai, ΔSi, Δθi and Δαi. Considering the working principle of the sensor, Δθi which is indirectly determined by other parameters has no realistic meaning in the course of error analysis.

If the position error and attitude error of the force-measuring platform are expressed as vectors $\Delta P={\left[\begin{array}{ccc} \Delta P_{x} & \Delta P_{y} & \Delta P_{z} \end{array}\right]}^{T}$ and $\Delta \delta ={\left[\begin{array}{ccc} \Delta \delta_{x} & \Delta \delta_{y} & \Delta \delta_{z} \end{array}\right]}^{T}$. Then, for the *i*-th leg, they can obtained as:

(13) {ΔPi=GaPi×Δai+GSPi×ΔSi+GαPi×ΔαiΔδi=GαRi×Δαi

If the influence of all legs’ error sources is taken into account, the vectors are rewritten as:

(14) {ΔP=16(∑i=16GaPi·Δai+∑i=16GSPi·ΔSi+∑i=16GαPi·Δαi)Δδ=16(∑i=16GαRi·Δαi)

Furthermore, the comprehensive position error and attitude error of the force-measuring platform are defined as:

(15) $$\{\begin{array}{l} \left|\Delta P\right|=\sqrt{{\left(\Delta P_{x}\right)}^{2}+{\left(\Delta P_{y}\right)}^{2}+{\left(\Delta P_{z}\right)}^{2}}\\ \left|\Delta \delta \right|=\sqrt{{\left(\Delta \delta_{x}\right)}^{2}+{\left(\Delta \delta_{y}\right)}^{2}+{\left(\Delta \delta_{z}\right)}^{2}} \end{array}$$

The sensor error sources analyzed in the above includes the radius errors of the force-measuring platform and fixed platform, errors of the two axial clearances for the lower positioning block and the middle universal joint and installation error of single-axis force sensor, which correspond to the five D-H parameters Δa67i, Δa01i, Δa12i, Δa45i and ΔS3i, respectively. According to the nine stage processing accuracy of the sensor, the tolerance ranges of each error source are respectively: TΔa67i=155 μm, TΔa01i=130 μm, TΔa12i=36 μm, TΔa45i=36 μm and TΔS3i=87 μm.

Now, the Monte Carlo simulation analysis method [29] is adopted to simulate and analyze the pose error of the force-measuring platform caused by assembly of 6-UPUR six-axis force sensor with flexible joints. Firstly, the error sources with different distribution characteristics are sampled. From the theory of mechanical technology, when the workpiece is produced in single batch and small-scale production, the dimension error is a normal distribution in its tolerance range T. According to ±3σ principle [30], standard deviation of each error source can be obtained as:

(16) σ=T6

Then the sampling value of these error sources is calculated by the following equation:

(17) $$\Delta W=\sigma \sqrt{-2ln\mu_{1}}\cos\left(2\pi \mu_{2}\right)$$

where both $\mu_{1}$ and $\mu_{2}$ are the random numbers between 0–1.

**Figure 8:**
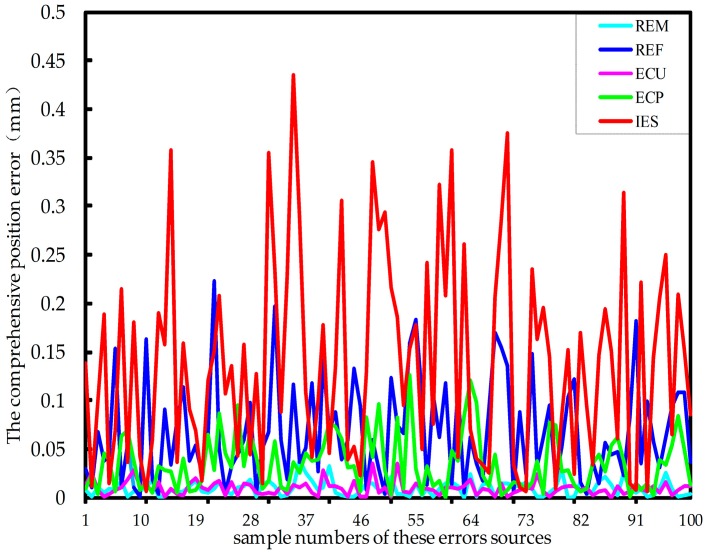
The influence of the five error sources on the comprehensive position error of the platform.

**Figure 9:**
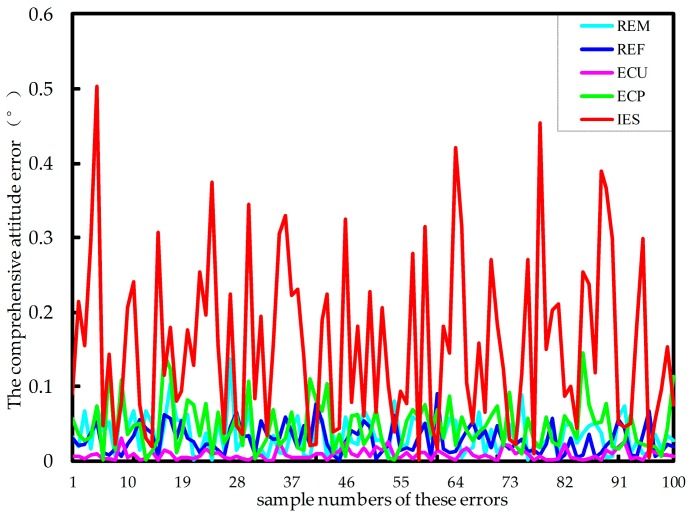
The influence of the five error sources on the comprehensive attitude error of the platform.

By MATLAB, the sample sizes of these error sources are all 100. Substituting in Equation (15), then the position error and attitude error are statistically simulated. Figure 8 and Figure 9 show the influence of all the five error sources on the comprehensive position error and the comprehensive attitude error of the force-measuring platform, respectively. It should be noted that in the legend, REM, REF, ECU, ECP and IES indicate the radius errors of the force-measuring platform and fixed platform, errors of two axial clearances for the middle universal joint and the lower positioning block and installation error of single-axis force sensor, respectively. 

It can be seen that the installation error of single-axis force sensor, among the five error sources, has the greatest influence on the comprehensive position and attitude error. Due to the cumulative amplification of errors, the radius error of the fixed platform and error of the two axial clearances on the lower positioning block also have great impact. Comparatively, the other two error sources have less impact. Meanwhile, the radius error of the force-measuring platform has a huge influence on the comprehensive attitude error. Therefore, conclusions can be drawn that the radius accuracy of force-measuring platform and fixed platform and axial mounting accuracy of single-axis force sensor particularly are ensured in the sensor manufacturing process.

## 4. Deformation Error Modeling of the 6-UPUR Force Sensor

In the working process of the sensor, the elastic deformation of flexible legs is objective. The actual working position of a reference point on the force-measuring platform will also change accordingly, which seriously affects the static performance of the sensor.

When a six-dimensional external force vector is exerted at the end of the *i*-th flexible series leg, it can be obtained as follows by the principle of virtual work:

(18) $$\{\begin{array}{l} \Delta S_{j}^{i}=J_{j}^{i}S_{j}^{i}={\left[\Delta x_{j}^{i},\Delta y_{j}^{i},\Delta z_{j}^{i},\Delta \alpha_{x_{j}}^{i},\Delta \alpha_{y_{j}}^{i},\Delta \alpha_{z_{j}}^{i}\right]}^{T}\\ F_{j}^{i}=J_{Fj}^{i}F^{i} \end{array}\left(\begin{array}{l} i=1,2,\cdots ,6\\ j=1,2,3,4 \end{array}\right)$$

where $\Delta S_{j}^{i}$ denotes the deformation vector at the end reference point caused by elastic deformation of the *j*-th basic flexible element for the *i*-th leg. $S_{j}^{i}$ stands for the elastic deformation vector produced by the end force $F^{i}$ at the end of the *j*-th basic flexible element for the *i*-th leg. $F_{j}^{i}$ refers to counterforce vector at the end of the *j*-th basic flexible element produced by the end force $F^{i}$. $J_{j}^{i}$ stands for the pose transformation matrix. $J_{Fj}^{i}$ denotes the force transformation matrix.

According to the superposition principle of deformation, the total deformation vector $\Delta S^{i}$ of the flexible leg end is obtained as follows:

(19) $$\Delta S^{i}=\sum_{j=1}^{4}\Delta S_{j}^{i}=\sum_{j=1}^{4}J_{j}^{i}S_{j}^{i}=J_{1}^{i}S_{1}^{i}+J_{2}^{i}S_{2}^{i}+\cdots +J_{6}^{i}S_{6}^{i}\left(i=1,2,\cdots ,6\right)$$

Under the definition of the stiffness matrix, the relationship between the leg end force $F^{i}$ and the total deformation vector $\Delta S^{i}$ is:

(20) $$F^{i}=K^{i}\Delta S^{i}\left(i=1,2,\cdots ,6\right)$$

where $K^{i}$ denotes stiffness matrix at the end of the flexible leg. Similarly, the counterforce vector $F_{j}^{i}$ at the end of the *j*-th basic flexible element can be expressed as:

(21) $$F_{j}^{i}=K_{j}^{i}\Delta S_{j}^{i}$$

Combining the above equations, the total deformation vector can be rewritten as:

(22) $$\Delta S^{i}={\left(K^{i}\right)}^{-1}F^{i}=\sum_{j=1}^{4}J_{j}^{i}\Delta S_{j}^{i}=\sum_{j=1}^{4}J_{j}^{i}{\left(K_{j}^{i}\right)}^{-1}F_{j}^{i}=\sum_{j=1}^{4}J_{j}^{i}{\left(K_{j}^{i}\right)}^{-1}J_{Fj}^{i}F^{i}\left(i=1,2,\cdots ,6\right)$$

where $K_{j}^{i}$ refers to the stiffness matrix of the *j*-th basic flexible element:

(23) $$K^{i}={\left(\sum_{j=1}^{4}J_{j}^{i}{\left(K_{j}^{i}\right)}^{-1}J_{Fj}^{i}\right)}^{-1}\left(i=1,2,\cdots ,6\right)$$

Then the stiffness matrix $K^{i}$ can be expressed easily. Based on the stiffness model of each leg, the overall stiffness matrix of flexible joints 6-UPUR six-axis force sensor can be obtained. At the same time, we assume that the force-measuring platform stiffness reaches infinity and the small deformation produced by the external force is ignored.

When a six-dimensional external force vector $F_{w}$ is exerted, the geometric compatibility condition between the end of the *i*-th leg and reference point of the force-measuring platform is as follows:

(24) ΔS=[ΔxΔyΔzΔαxΔαyΔαz]=[RTOipOp−RTOipOpS(ri)03×3RTOipOp]·[ΔxiΔyiΔziΔαxiΔαyiΔαzi]=JiΔSi

where ΔS stands for the deformation vector at the center reference point of the force-measuring platform.

Δx and $\Delta x^{i}$ refer to the linear displacement vector of the force-measuring platform and the *i*-th leg along x-axis, respectively. Similarly, Δy, $\Delta y^{i}$, Δz and $\Delta z^{i}$ denote those along the y-, and z-axis, respectively. $\Delta \alpha_{x}$ and $\Delta \alpha_{x}^{i}$ refer to the angular displacement vector of the force-measuring platform and the *i*-th leg along x-axis, respectively. Similarly, $\Delta \alpha_{y}$, $\Delta \alpha_{y}^{i}$ $\Delta \alpha_{z}$ and $\Delta \alpha_{z}^{i}$ denote those along the y-, and z-axis, respectively. ROipOp stands for the rotation matrix of the measuring platform expressed in a local coordinate frame where the moving coordinate frame $\left\{O_{p}\right\}$ is relative to the local coordinate frame $\left\{O_{ip}\right\}$. $S\left(r_{i}\right)$ refers to the vector of the platform expressed in the fixed coordinate frame.

According to the principle of spatial force system synthesis, the relationship between the six-dimensional external force vector $F_{w}$ and the counterforce vector $F^{i}$ at the end of the *i*-th leg can be established as:

(25) Fw=[fxfyfzmxmymz]=∑i=16([ROipOp03×3S(ri)ROipOpROipOp][fxifyifzimximyimzi])=∑i=16JFiFi

In addition, according to the definition of stiffness matrix of the flexible parallel mechanism, the six- dimensional external force vector $F_{w}$ is:

(26) $$F_{w}=K\Delta S=\sum_{i=1}^{6}J_{F}^{i}F^{i}=\sum_{i=1}^{6}J_{F}^{i}K^{i}\Delta S^{i}=\sum_{i=1}^{6}J_{F}^{i}K^{i}{\left(J^{i}\right)}^{-1}\Delta S$$

Then the stiffness matrix K of the reference point is expressed as:

(27) $$K=\sum_{i=1}^{6}J_{F}^{i}K^{i}{\left(J^{i}\right)}^{-1}$$

When an external force $F_{w}$ exerted on the platform changes by $\delta F_{w}$, the micro displacement vector of the reference point is:

(28) $$\delta D=K^{-1}\delta F_{w}$$

Then, the deformation error of the platform caused by elastic deformation of the flexible legs can be solved by Equation (28) when the external force $F_{w}$ exerted on the platform changes. When the external force $f_{w}$ or the torque $m_{w}$ exerted on the platform change by 1000 N or 1000N m, the corresponding deformation vectors calculated by Equation (28) are shown as Table 2.

## 5. Synthetic Error of the 6-UPUR Parallel Six-Axis Force Sensor

Assume the assembly error and deformation error are expressed as $\Delta D_{1}$ and $\Delta D_{2}$, respectively. $\Delta D_{1}$ is obviously a function with respect to the sensor structure parameters, which is certain for the processed sensor. When the external force exerted on the platform is certain, that is to say, $\Delta D_{2}$ is assured, then, the synthetic error ΔD of the platform is the deformation coupling resulting from the assembly and exerted force, which can expressed as:

(29) $$\Delta D=\Delta D_{1}+\Delta D_{2}=\left[\begin{array}{cccccc} \Delta P_{x} & \Delta P_{y} & \Delta P_{z} & \Delta \delta_{x} & \Delta \delta_{y} & \Delta \delta_{z} \end{array}\right]$$

As is well known, when the synthetic error of the platform is taken into account, the homogeneous transformation matrix with respect to ideal position of the platform is:

(30) $$\Delta T=\left[\begin{array}{cc} \Delta R & \Delta P\\ 0_{1\times 3} & 1 \end{array}\right]$$

where ΔP is translational component of the force-measuring platform, $\Delta P={\left[\begin{array}{ccc} \Delta P_{x} & \Delta P_{y} & \Delta P_{z} \end{array}\right]}^{T}$; ΔR can be expressed by the RPY description method:

$$\Delta R=\left[\begin{array}{ccc} \cos\Delta \delta_{z}\cos\Delta \delta_{y} & \sin\Delta \delta_{z}\cos\Delta \delta_{y} & -\sin\Delta \delta_{y}\\ \cos\Delta \delta_{z}\sin\Delta \delta_{y}\sin\Delta \delta_{x}-\sin\Delta \delta_{z}\cos\Delta \delta_{x} & \sin\Delta \delta_{z}\sin\Delta \delta_{y}\sin\Delta \delta_{x}+\cos\Delta \delta_{z}\cos\Delta \delta_{x} & \cos\Delta \delta_{y}\sin\Delta \delta_{x}\\ \cos\Delta \delta_{z}\sin\Delta \delta_{y}\cos\Delta \delta_{x}+\sin\Delta \delta_{z}\sin\Delta \delta_{x} & \sin\Delta \delta_{z}\sin\Delta \delta_{y}\cos\Delta \delta_{x}-\cos\Delta \delta_{z}\sin\Delta \delta_{x} & \cos\Delta \delta_{y}\cos\Delta \delta_{x} \end{array}\right]$$

Then, the transform matrix of the force-measuring platform after deformation is:

(31) $$T=\Delta TT_{0}=\left[\begin{array}{cc} R^{\prime } & P^{\prime }\\ 0_{1\times 3} & 1 \end{array}\right]$$

where $T_{0}$ represents the pose transformation matrix of the ideal position of the force-measuring platform expressed in the fixed coordinate frame.

Here, taking into account the synthetic error, $G^{\prime }$ can be calculated by Equation (32):

(32) $$G^{\prime }=\left[\begin{array}{llll} S_{1}^{\prime } & S_{2}^{\prime } & \cdots & S_{6}^{\prime }\\ S_{01}^{\prime } & S_{02}^{\prime } & \cdots & S_{06}^{\prime } \end{array}\right]$$

## 6. Deformation Measurement and Calibration Experiments

This experimental equipment consists of a hardware and software system. The former mainly includes a hydraulic loading system, loading calibration bench, signal processing device, data acquisition device, data processor, etc. The hydraulic loading system provides the loading force. By calibrating the two hydraulic cylinders in the loading calibration bench, which transmit force to the measuring platform, and adjusting the installation positions of the two loading units every time, six dimensional forces and torques can be exerted on the platform. There are eight output signal channels from the single-axis tension-compression sensor when the calibration experiments are performed. The signals are transmitted to the computer by a signal processing device and data acquisition card, and then processed by the calibration software system. 

In the loading process of the deformation measurements, one or two loading units should be chosen according to the loading direction. The specific implementation is as follows: a loading unit is installed on one upright column side along the $X_{B}$-axis. By adjusting the tension/compression mode of the hydraulic cylinder, the loading force along the $X_{B}$-axis can be achieved. The same is true of the loading along the $Y_{B}$-axis. Both loading units are installed on two upright column ends in the direction of the $X_{B}$-axis, then the loading force along the $Z_{B}$-axis can be achieved. Both loading units are installed on two upright column sides along the $Y_{B}$-axis. By adjusting the tension/compression mode of the hydraulic cylinder, the loading torque along the $X_{B}$-axis can be achieved. Similarly, both loading units are installed on two upright column sides in the direction of the $X_{B}$-axis, and then the loading torque along the $Y_{B}$-axis can be obtained. Two loading units are installed on two upright column different sides in the direction of the $X_{B}$-axis or the $Y_{B}$-axis, respectively. Then the loading torque along the $Z_{B}$-axis can be obtained.

**Figure 10:**
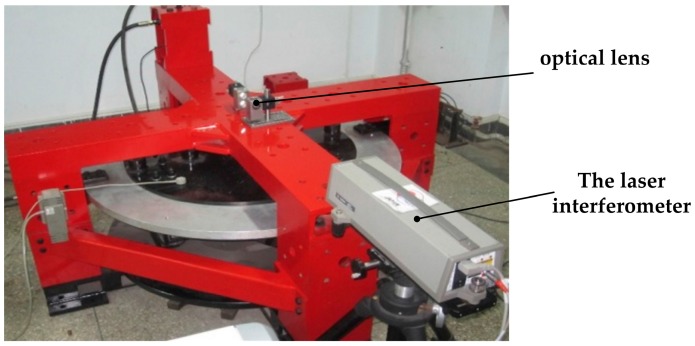
Laser interferometer and optical lens installation location.

**Figure 11:**
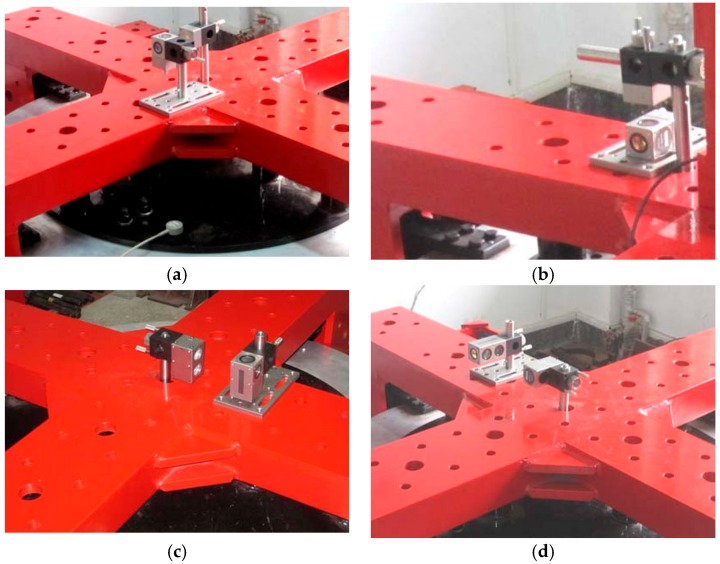
Optical lens installation measuring in six directions. (**a**) Linear displacement measurement along the $X_{B}/Y_{B}$-axis; (**b**) Linear displacement measurement along the $Z_{B}$-axis; (**c**) Pitching angle measurement around the $X_{B}/Y_{B}$-axis; (**d**) Swing angle measurement around the $Z_{B}$-axis.

Based on the loading location of force and torque mentioned above, an optical lens is mounted on the measuring platform. The position of a laser interferometer is adjusted and then the deformation of the platform can be measured. The laser interferometer and optical lens installation location are shown in Figure 10 and Figure 11, respectively.

Each axial force/torque within the sensor range is divided into 10 load points in two positive and negative directions, respectively, as shown in Table 3. Load force or torque in a corresponding direction are applied according to the positive direction of loading points. Conversely the reversely load is applied in descending order. Then, we save the data of the laser interferometer loaded every time. We follow the experimental steps described in [12], and then check and process the data and decoupled calculation and result analysis are carried out.

### 6.1. Measurement Results and Analysis

**Figure 12:**
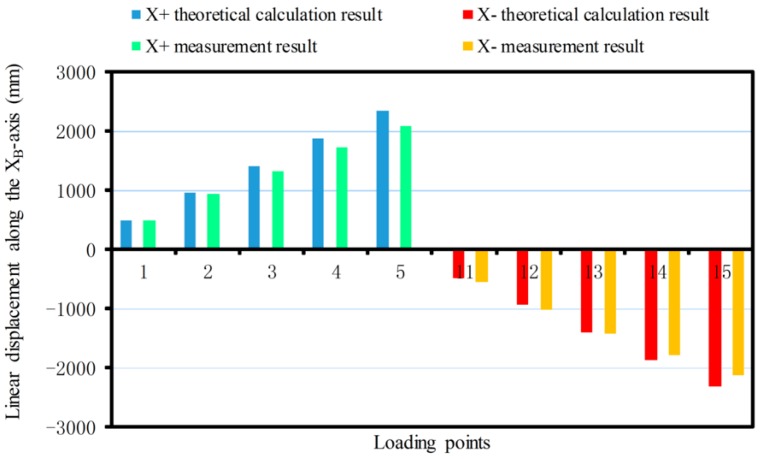
Linear displacement comparison along the $X_{B}$-axis.

**Figure 13:**
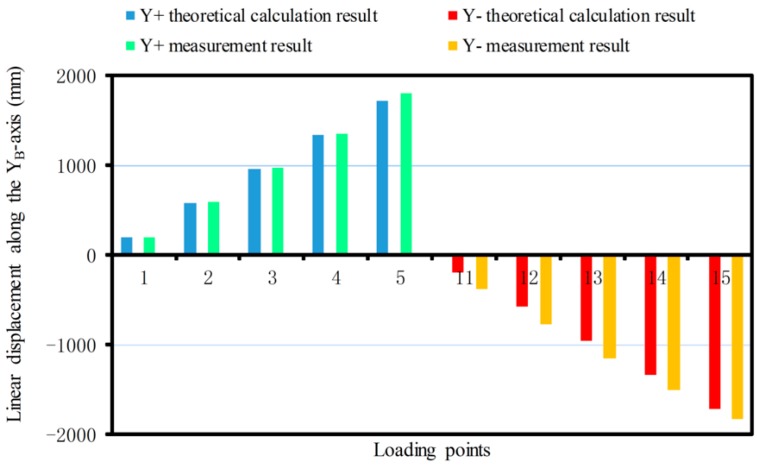
Linear displacement comparison along the $Y_{B}$-axis.

**Figure 14:**
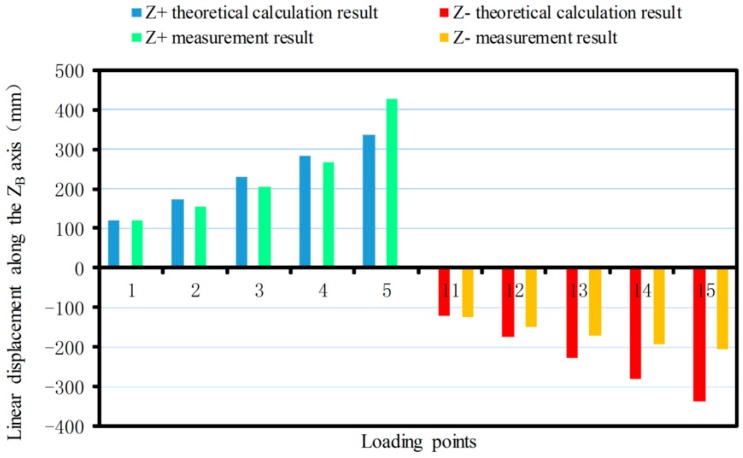
Linear displacement comparison along the $Z_{B}$-axis.

**Figure 15:**
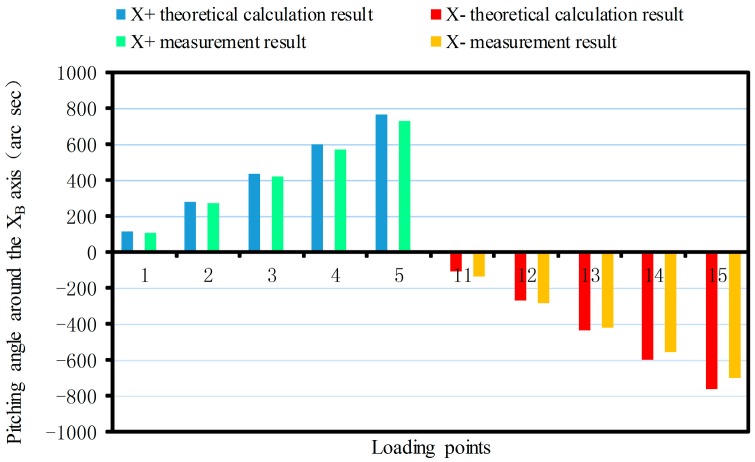
Pitching angle comparison around the $X_{B}$-axis.

**Figure 16:**
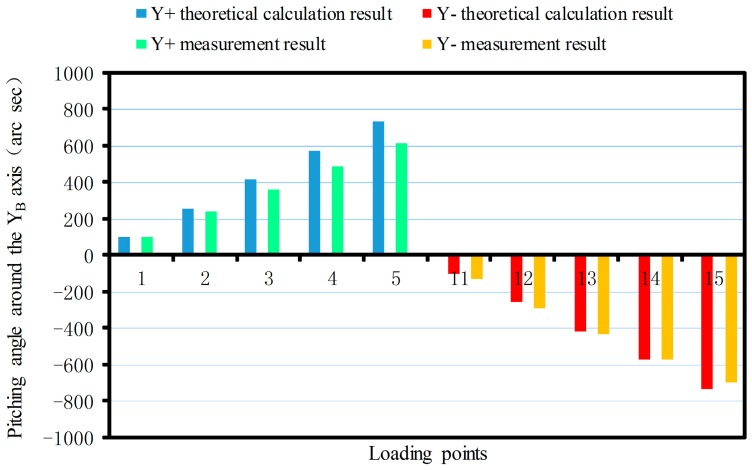
Pitching angle comparison around the $Y_{B}$-axis.

**Figure 17:**
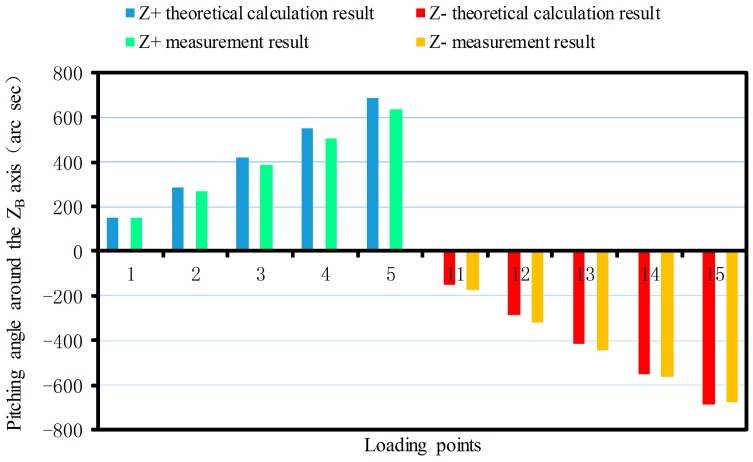
Pitching angle comparison around the $Z_{B}$-axis.

The linear displacement or pitching angle comparisons of the platform between the calibration deformation measurement results and the theoretical calculation results of the synthetic error are made as shown in Figure 12, Figure 13, Figure 14, Figure 15, Figure 16 and Figure 17.

Since the sensor structure is theoretically symmetrical about the $X_{B}$-axis, so in the theoretical calculation, when the force is exerted along the $X_{B}$-axis, the linear displacement along the positive and negative half of the $X_{B}$-axis is symmetrical about the $X_{B}$-axis, and with any increase of the loading force, the linear displacements along the positive and negative half of the $X_{B}$-axis are linearly increased. The theoretical calculation values of the maximum displacement are 2327.3 μm and −2327.3 μm, respectively. The maximum positive and negative measurements are 2079.88 μm and −2129.72 μm as shown in Figure 12.

Similarly, the theoretical calculation value of the maximum displacement along positive and negative half of the $Y_{B}$-axis are 1714.85 μm and −1714.85 μm, respectively. The maximum measurements are 1799.87 μm and −1838.26 μm (Figure 13). The theoretical value of the maximum displacement along the positive and negative $Z_{B}$-axis are 336.5 μm and −336.5 μm, respectively. The maximum measurements are 428.21 μm and −206.99 μm (Figure 14).

As shown in Figure 15 since the sensor structure is symmetrical theoretically about the $X_{B}$-axis with the increase of the loading torque, the theoretical calculation values of the maximum pitching angle around the $X_{B}$-axis are 766.2 arc s and −766.2 arc s. The maximum positive and negative measurements are 729.4 arc s and −700.57 arc s, respectively. For the same reason, the theoretical calculation value of the maximum pitching angle around the positive and negative $Y_{B}$-axis are 731.4 arc s and −731.4 arc s, respectively. The maximum measurements are 612.75 arc s and −697.37 arc s (Figure 16). The theoretical value of the maximum pitching angle around the positive and negative $Z_{B}$-axis are 685.75 arc s and −685.75 arc s, respectively. The maximum measurements are 635.94 arc s and −674.47 arc s (Figure 17).

From Figure 17, it can be seen that when the force is exerted along the $Z_{B}$-axis, deformation of the force-measuring platform has an obvious nonlinear relationship with the magnitude of the force, and the deviation is larger, compared with the theoretical result. The main reason is that the loading force along the $Z_{B}$-axis is achieved by two loading units, which are installed at both ends of the loading benches along the $X_{B}$-axis, rather than loading the platform directly along the $Z_{B}$-axis as in the theoretical analysis. Because of manufacturing errors, it is difficult to achieve complete symmetry of the sensor structure, so the measurement value will produce a deviation with the theoretical value. When the force/torque is exerted along the other directions, the deformation of the force-measuring platform basically has a linear relationship with the magnitude of the force/torque, and the measured results are basically consistent with the theoretical results. Then, the correctness of synthetic error model is verified. At the same time, the deformation error of the flexible leg is the main error factor that affects sensor accuracy and with increase of the loading force/torque, so the proportionality is more obvious.

### 6.2. Calibration Results and Analysis

In this section, the actual first order kinematic influence coefficient matrix $G_{B}^{\prime }$ is obtained by the calibration results. Then we compare it with theoretical first order kinematic influence coefficient matrix $G_{}^{\prime }$ and the first order kinematic influence coefficient matrix G when the synthetic error is taken into account.

The relationship between external force and output voltage matrix is $F_{w}=G_{B}V$. Then the calibration matrix can be expressed as $G_{B}=F_{w}V^{T}{\left(VV^{T}\right)}^{-1}$ by the least squares method [14]. Next, $G_{B}$ will be transformed into the transfer relation matrix between the external force and the measuring force, that is, the actual first order kinematic influence coefficient matrix $G_{B}^{\prime }$.

From the technical parameters of the force sensitive element, the spokewise single-axis force sensor, it can be known that its range is 2 t; the sensitivity is 2.0±0.01mV/V and supply voltage is DC10 V, so when the sensor is loaded by 2 t, the output signal of the sensor is 20mV.

Assume that $f_{a}^{\prime }$ represents the actual axial force of the single-axis force sensor, whose units are N or N m. The actual output signal of the single-axis sensor is expressed as $V^{\prime }$, whose units are mV. Here, the relationship between them is $V^{\prime }=f_{a}^{\prime }/980$. On the other hand, due to circuit amplification and denoising, the relationship between V and $V^{\prime }$ is $V=kV^{\prime }$ (k stands for voltage amplification factor). Therefore, the transfer relationship between the external force and the actual axial force is:

(33) $$F_{w}=G_{B}V=kG_{B}V^{\prime }=\frac{k}{980}G_{B}f_{a}^{\prime }$$

Afterwards, the actual first order kinematic influence coefficient matrix $G_{B}^{\prime }$ is:

(34) $$G_{B}^{\prime }=\frac{k}{980}G_{B}$$

So far, the theoretical first order kinematic influence coefficient matrix G, the first order kinematic influence coefficient matrix $G_{}^{\prime }$ when the synthetic error is taken into account and the actual first order kinematic influence coefficient matrix $G_{B}^{\prime }$ can be calculated easily by Equations (4), (32) and (34), respectively. As is well known the condition number [31] of the first order kinematic influence coefficient matrix is one of the indices to measure isotropy for a force sensor. Consequently, their condition numbers are calculated as:

(35) $$\{\begin{array}{l} cond\left(G\right)=6.3336\\ cond\left(G^{\prime }\right)=11.7549\\ cond\left(G_{B}^{\prime }\right)=10.7808 \end{array}$$

From Equation (35), it can be seen that condition number of $G_{}^{\prime }$ is more close to that of $G_{B}^{\prime }$ than G’s. By calculation, the relative errors are 9.03% and 41.25%, respectively. Obviously, $G_{}^{\prime }$ is similar to $G_{B}^{\prime }$.

On the other hand, the square norm [27] of a channel output signal vector is used to measure sensitivity of a generalized force component. The sensitivity $S_{Fx}$, $S_{Fy}$ and $S_{Fz}$ of the three force components can be expressed as:

(36) $$\left\{ \begin{array}{l} S_{Fx}={\left\|J_{1}\right\|}_{2}\\ S_{Fy}={\left\|J_{2}\right\|}_{2}\\ S_{Fz}={\left\|J_{3}\right\|}_{2} \end{array}\right.$$

The sensitivity $S_{Mx}$, $S_{My}$ and $S_{Mz}$ of the three torque components are expressed as:

(37) $$\left\{ \begin{array}{l} S_{Mx}={\left\|J_{4}\right\|}_{2}\\ S_{My}={\left\|J_{5}\right\|}_{2}\\ S_{Mz}={\left\|J_{6}\right\|}_{2} \end{array}\right.$$

where $J_{i}(i=1,2,\cdots ,6)$ stands for column vector of force Jacobian matrix.

All the component sensitivities of the three force Jacobian matrices are calculated as shown in Table 4. It can be seen that sensitivity of $J_{}^{\prime }$ is more close to that of $J_{B}^{\prime }$ than J’s. Their square norm relative errors can be seen in Table 5. Here their relative errors are defined as Type 1 error and Type 2 error.

Table 5 shows that the Type 1 error is less than the Type 2 error. That is to say, $G_{}^{\prime }$ is more close to $G_{B}^{\prime }$ than G. It is worth noting that Type 1 $S_{Fz}$ has a larger relative error. The main reason is that the two loading units are installed at both ends of loading benches along the $X_{B}$-axis to provide the $Z_{B}$-axis loading force, which is explained in the previous section. Consequently, we will not bore readers with a very detailed analysis to explain the reason any more. Obviously, the effectiveness of the error model is clarified and it is very important to take into account synthetic errors for the design stage of the sensor and this is helpful to improve the performance of the sensor in order to meet the needs of actual working environments.

## 7. Conclusions

In this paper, assembly error and deformation error are comprehensively taken into account based on the flexible joints 6-UPUR parallel six-axis force sensor developed with a large measurement range and high accuracy in the prophase. Their error models are respectively established. The synthetic error of the platform is deformation coupling resulting from assembly and exerted force. Then the first order kinematic influence coefficient matrix when the synthetic error is taken into account is solved. Measurements and calibration experiments are carried out. Forced deformation of the force-measuring platform is detected by using a laser interferometer and analyzed to verify the correctness of the synthetic error model. In addition, the first order kinematic influence coefficient matrix in actual circumstances is calculated. Condition numbers and square norms of the coefficient matrices are compared, which shows theoretically that it is very important to take into account the synthetic error for the design stage of the sensor and this is helpful to improve the performance of the sensor in order to meet needs of actual working environments.

## Figures and Tables

**Table 1:** Material properties of the sensor.

Components	Materials	Elastic Modulus	Poisson Ratio	Density
Force-measuring platform	Hard aluminum alloy	70 Gpa	0.30	2700 kg/m^3^
Flexible joints	40CrNiMoA	206 Gpa	0.30	7830 kg/m^3^
Fixed platform	Q235	210 Gpa	0.25	7850 kg/m^3^

**Table 2:** Theoretical calculation value of reference point deformation of the force-measuring platform.

Force/Torque Variation (N/N·m)	Force along X Axis	Force along Y Axis	Force along Z Axis	Torque around X Axis	Torque around Y Axis	Torque around Z Axis
1000	230 μm	190 μm	27 μm	82 arc s	79 arc s	67 arc s

**Table 3:** Loading points of calibration force/torque.

Loading Points		1	2	3	4	5	6	7	8	9	10
		11	12	13	14	15	16	17	18	19	20
**Force (N)**	**positive**	1000	3000	5000	7000	9000	7000	5000	3000	1000	0
	**negative**	−1000	−3000	−5000	−7000	−9000	−7000	−5000	−3000	−1000	0
**Torque (N·m)**	**positive**	1000	3000	5000	7000	9000	7000	5000	3000	1000	0
	**negative**	−1000	−3000	−5000	−7000	−9000	−7000	−5000	−3000	−1000	0

**Table 4:** All the component sensitivities of the three force Jacobian matrices.

Sensitivity	$S_{Fx}$	$S_{Fy}$	$S_{Fz}$	$S_{Mx}$	$S_{My}$	$S_{Mz}$
J	0.2964	0.5472	0.5473	1.5025	1.3973	1.3974
$J^{\prime }$	1.0459	0.8465	0.9009	6.8417	3.7478	6.0042
$J_{B}^{\prime }$	1.4205	0.9930	1.8518	8.2074	3.2378	6.9202

**Table 5:** The two type relative errors of all the component sensitivity.

Sensitivity	$S_{Fx}$ (%)	$S_{Fy}$ (%)	$S_{Fz}$ (%)	$S_{Mx}$ (%)	$S_{My}$ (%)	$S_{Mz}$ (%)
Type 1 error	26.37	14.75	51.35	16.64	15.75	13.24
Type 2 error	79.13	44.89	70.44	81.69	56.84	79.81
